# Eukaryotic-type serine/threonine kinase mediated phosphorylation at Thr^169^ perturbs mycobacterial guanylate kinase activity

**DOI:** 10.1042/BSR20171048

**Published:** 2017-11-15

**Authors:** Ghanshyam S. Yadav, Sandeep K. Ravala, Sangita Kachhap, Meghna Thakur, Abhishek Roy, Balvinder Singh, Subramanian Karthikeyan, Pradip K. Chakraborti

**Affiliations:** CSIR-Institute of Microbial Technology, Sector 39A, Chandigarh 160 036, India

**Keywords:** enzyme kinetics, mass spectrometry, molecular dynamic simulations, phosphorylation/dephosphorylation, site-directed mutagenesis

## Abstract

Guanylate kinase is an essential and conserved enzyme in nucleotide biosynthetic pathway that transfers phosphoryl group of ATP to GMP for yielding GDP. Here, we report the phosphorylation of guanylate kinase from *Mycobacterium tuberculosis* (mGmk) by eukaryotic-type Ser/Thr kinase, PknA. Mass spectrometric studies identified Thr^101^ and Thr^169^ as phosphorylatable residues in mGmk. To evaluate the significance of phosphorylation in these threonines, two point (T101A and T169A) and one double (T101A-T169A) mutants were generated. The kinase assay with these mutant proteins revealed the major contribution of Thr^169^ compared with Thr^101^ in the phosphorylation of mGmk. Kinetic analysis indicated that p-mGmk was deficient in its enzymatic activity compared with that of its un-phosphorylated counterpart. Surprisingly, its phosphoablated (T169A) as well as phosphomimic (T169E) variants exhibited decreased activity as was observed with p-mGmk. Structural analysis suggested that phosphorylation of Thr^169^ might affect its interaction with Arg^166^, which is crucial for the functioning of mGmk. In fact, the R166A and R166K mutant proteins displayed a drastic decrease in enzymatic activity compared with that of the wild-type mGmk. Molecular dynamics (MD) studies of mGmk revealed that upon phosphorylation of Thr^169^, the interactions of Arg^165^/Arg^166^ with Glu^158^, Asp^121^ and residues of the loop in GMP-binding domain are perturbed. Taken together, our results illuminate the mechanistic insights into phosphorylation-mediated modulation of the catalytic activity of mGmk.

## Introduction

Nucleotide metabolism is a crucial process in all spheres of life. Nucleotides comprise of both purine and pyrimidine bases and they are required for different cellular processes [1]. While pyrimidine synthesis is responsible for generation of CTP/UTP, metabolism of purine nucleotide involves generation of ATP/GTP, the energy currency of the cell. Both ATP and GTP are required by the cells to perform essential functions, which is evident by the involvement of ATPases and GTPases in most of the cellular processes [1]. In fact, any alteration in synthesis of these molecules may affect proper functioning of a cell [2]. GTP also plays a key role in the synthesis of pyrimidine nucleotides and dicyclic GMP [3,4]. Furthermore, GTP pool in bacteria is associated with stress response by forming (p)ppGpp that regulates purine (GTP) biosynthesis [5]. Available reports also indicated that high levels of GTP cause bacterial cell death [5]. In *Salmonella typhimurium*, mutants of purine synthesis pathway exhibited defective growth phenotype in human serum [6]. Therefore, requirement of nucleotides in bacteria is known to be critical, especially in pathogens during infection to host [7]. Previously, regulation of purine metabolism by eukaryotic-type Ser/Thr kinase mediated phosphorylation in *Streptococcus agalactiae* was reported [8]. In this context, we concentrated on *Mycobacterium tuberculosis*, an intracellular pathogen causing tuberculosis, a disease responsible for considerable human mortality worldwide [9].

Available literature indicated that 11 eukaryotic-type Ser/Thr kinases are present in *M. tuberculosis* and they are involved in catalysing dynamic phosphotransfer reactions. Together with the single cognate phosphatase, these kinases operate in a concerted manner in regulating several metabolic functions in this bacteria [10]. In fact, *M. tuberculosis*, grown in different conditions exhibited 516 phosphorylation events in 301 proteins, which is the highest number reported for any bacteria [10,11]. Among them, *M. tuberculosis* guanylate kinase (mGmk), an essential enzyme belonging to NMP kinase family of proteins, was identified as a phosphorylatable substrate of mycobacterial eukaryotic-type Ser/Thr kinases through genome-wide phosphoproteome mapping [11]. However, the consequences of its phosphorylation are not known till date. It is well known that guanylate kinase in bacteria is a crucial enzyme in nucleotide biosynthetic pathway involved in converting GMP into GDP by transferring phosphoryl group from ATP. We were therefore, interested to evaluate whether phosphosphorylation has any effect on the activity of mGmk. For this, we utilized PknA, a representative of eukaryotic-type Ser/Thr kinases present in *M. tuberculosis*, which is reported to be associated with cell division as well as in regulating several metabolic processes [12–18]. *PknA* is an essential gene [19], and also present in the minimal genome of *Mycobacterium leprae* [20].

In the present study, we report that mGmk is transphosphorylated by PknA. MS studies together with mutational analysis revealed that Thr^169^ of mGmk is the major phosphosite. Structural analysis along with molecular simulation data further indicated that phosphorylation of Thr^169^ would prevent interaction of Arg^165^/Arg^166^ with Glu^158^, Asp^121^ and residues (from 92–96) of loop in GMP domain thereby affecting the catalytic functioning of mGmk.

## Materials and methods

### Constructs and site-directed mutagenesis

We noticed 100% identity of *M. tuberculosis gmk* gene (*m-gmk*; Rv1389) sequences between its avirulent (H37Ra) and virulent (H37Rv) strains. Therefore, *M. tuberculosis* genomic DNA used in the present study was isolated from avirulent strain using a commercially available kit following the manufacturer’s protocol. The *m-gmk* gene was amplified by employing PCR (denaturation: 5 min at 95°C; reaction: 1 min at 95°C, 0.5 min at 58.7°C, 0.5 min at 72°C for 29 cycles; final extension: 10 min at 72°C) using gene-specific primers (CG1: forward 5′-CGATTCGAATTCCATATGAGCGTCGGCGAG-3′ and CG2: reverse 5′-ATTACAAGCTTTCATGGGGAGCCCG-3′; final concentration of each primer =1 µM/reaction), dNTP (100 µM), Herculase fusion DNA polymerase (Stratagene, 0.5 µl/50 µl total reaction volume) and *M. tuberculosis* genomic DNA (100 ng) as the template. The amplified gene fragments were ligated into pET28c and pVV2 vectors at the NdeI/HindIII sites resulting in pET-mGmk and pVV2-mGmk plasmids respectively. The ligated products were transformed in *Escherichia coli* strain DH5α for its amplification. Cloning of gene was ensured by restriction digestion of the plasmids prepared from the transformants and finally confirmed by sequencing in an automated DNA sequencer (Applied Biosystems). The PknA (pMAL-PknA), PknA-core (pMAL-PknA-338), kinase-dead mutant (pMAL-PknA-K42N), PknB (pMAL-PknB), PPP (pMAL-PPP) and PPP-G117D (pMAL-PPP-G117D) constructs used in the present study were described elsewhere [18,21,22].

Different point mutants of mGmk (T101A/T169A/R166K/R166A) and a double mutant (-T101A/T169A) were generated using PCR overlap extension method [23], where two external primers (CG1/CG2) and two internal primers incorporating desired mutations (T101A: 5′-CTGCACCGGTCAGGAGCTTTGGCCCAGCCG-3′/5′-CGGCTGGGCCAAAGCTCCTGACCGGTGCAG-3′; T166A: 5′-GTTATCCAACGAGCCCTCGACACCGCGCGG-3′; R166A: 5′-GTTATCCAACGAGCCCTCGACACCGGCGG-3′/5′-CCGCGCGGTGTCGAGGGCTCGTTGGATAAC-3′; R166K: 5′-GTTATCCAACGAAAACTCGACACCGCGCGG-3′/5′-CCGCGCGGTGTCGAGTTTTCGTTGGATAAC-3′; T169A: 5′-GCCTGGACGCCGCGCGGATCG-3′/5′-CGATCCGCGCGGCGTCCAGGC-3′; T169E: 5′-GCCTGGACGAAGCGCGGATC-3′/5′-GATCCGCGCTTCGTCCAGGC-3′) were used. Final PCR reaction product(s) containing desired mutation(s) were ligated in pET28c vector and transformed in *E. coli* strain DH5α for screening of mutant clones.

### Expression and purification of recombinant proteins

*E. coli* strain BL21 (DE3) or TB1 cells were used for expression of recombinant proteins. The overnight cultures (14 h at 37°C) of transformed *E. coli* cells with different constructs were re-inoculated (1% inoculum) in fresh LB medium supplemented with antibiotics (50 µg/ml kanamycin and 100 µg/ml ampicillin for pET28c and for pMAL respectively), grown till OD_600_ of 0.6 and then induced with 0.4 mM IPTG (3 h at 37°C). Cells were harvested, resuspended in lysis buffer (50 mM Tris buffer, pH 7.5 containing 150 mM NaCl for His-tagged protein or 20 mM Tris buffer, pH 7.5 containing 200 mM NaCl for MBP-tagged protein and supplemented with 1 mM PMSF, 1 μg/ml pepstatin and 1 μg/ml leupeptin) and sonicated for 10 min (amplitude: 20%, frequency: 10 s ‘on’ and 15 s ‘off’) at 4°C. For purification of His-tagged proteins, supernatant fraction was loaded on an Ni-NTA column, washed with 20 ml of lysis buffer containing 10 mM imidazole and eluted in elution buffer (1 ml of lysis buffer containing 100 mM imidazole). Imidazole from the protein preparations was usually not removed since its presence did not affect the enzyme activity. The MBP-tagged protein was purified using an amylose column and eluted with 10 mM maltose following manufacturer’s (New England Biolabs, U.S.A.) recommended protocol. Protein concentrations of eluted samples were estimated by Bradford method [24] and stored in aliquots at −80°C until used for assays. To obtain p-mGmk protein, *E. coli* strain BL21 (DE3) cells were co-transformed with pET-mGmK and pMAL-PknA or p19Kpro-PknA, purified as mentioned above and was utilized for carrying out enzyme activity or MS studies. Culture of *Mycobacterium smegmatis* strain mc^2^155, transformation of pVV-mGmk and purification of recombinant protein is described elsewhere [25].

### Kinase and phosphatase assays

Transphosphorylation of mGmk or its mutants (T101A, T169A and T101AT169A) by PknA/PknA-core or PknB was monitored by performing *in vitro* kinase assays following the method reported previously [21]. Briefly, in each kinase reaction (usually 20 μl) PknA/PknA (1-338)/PknA K42N (1 µg) or PknB (1 µg), kinase buffer (50 mM Tris-Cl, pH 7.5, 50 mM NaCl, 10 mM MnCl_2_) and 2 μCi of [γ-^32^P] ATP (50 µM/reaction; effective radiolabelled ATP concentration of the stock solution is 2 mCi/μmole, obtained from Jonaki Laboratories, Board of Radiation and Isotope Technology, Hyderabad, India) was incubated with or without mGmk or its mutants for 30 min at 25°C and reaction was terminated by adding SDS sample buffer (5% v/v glycerol, 30 mM Tris-Cl, pH 6.8, 2.5% v/v β-mercaptoethanol, 1% w/v SDS and 0.01% w/v Bromophenol Blue). The samples were boiled for 5 min and separated on SDS/PAGE (12% gel). The gel was stained with Coomassie Brilliant Blue, analysed in a phosphoimaging device (Fuji Film model FLA 9000/Bio–Rad) and processed for autoradiography by exposing to Kodak X-Omat/AR film. In assessing dephosphorylation activity, phosphorylated proteins following incubation with PPP or its variant (25°C for 1 h), were processed as described elsewhere [18].

### Western blotting

Protein samples resolved on SDS/PAGE (12% gel) were transferred on to nitrocellulose membrane (0.45 µm) using Bio–Rad mini-transblot apparatus (120 V for 1 h). Following transfer, blots were incubated in blocking solution of 5% BSA for an hour and washed with TBS supplemented with 0.1% Tween 20 (TBST) four times for 15 min each. Blots were probed with primary antibodies (1:1000 dilution of anti-p-threonine (anti-p-Thr) with overnight incubation at 4°C diluted in blocking solution or for 1:3000 dilution of anti-His for 1 h at 25°C). Blots were washed four times with TBST (15 min/wash) and incubated with the horseradish peroxidase labelled secondary antibody (1:5000 dilution; anti-rabbit for anti-p-Thr and anti-mouse for anti-His antibodies). Finally, the blot(s) was developed using Luminataforte™ (Millipore) following manufacturer’s recommended protocol and signal was captured by exposing to X-ray film (Kodak, U.S.A.).

### Enzyme activity assay

Purified mGmk and different mutant proteins prepared from *E. coli* strain BL21 (DE3) cells harbouring pET-mGmk, pET-mGmk-T169A, pET-mGmk-T169E, pET-mGmk-R166K, pET-mGmk-R166A were used for the activity assay. Enzymatic activity of unphosphorylated (mGmk) or p-mGmk and different mutant proteins were determined using the coupled spectrophotometric assay (30°C/340 nm) in a microplate reader (Spectramax plus 384, Molecular Devices or Synergy Plate Reader, BioTek) [26]. The reaction mixture (final volume =200 µl) contained 50 mM Tris/HCl, pH 7.5, 0.2–0.3 mM NADH, 0.5 mM phosphoenol pyruvate, 50 mM KCl, 2 mM MgCl_2_, 400 µM ATP, 1 unit each of lactate dehydrogenase and pyruvate kinase, and 200 ng protein with varying concentrations of GMP (0–500 µM). The absorbance was measured every 10–20 s till OD reached near baseline. Specific activity of this enzyme was determined as described elsewhere [27]. Unless mentioned otherwise, the experiments in the present study were done at least three times and data represented as mean ± S.D.

### MS

Peptides were generated from phosphorylated and unphosphorylated forms of His-tagged mGmk proteins in solution by trypsin digestion at 37°C for 16 h using 100 mM ammonium bicarbonate buffer, pH 8.5. The samples were then processed for LC MS/MS as described elsewhere [18] and the machine readouts were analysed utilizing ProteinPilot™ software (http://www.absciex.com/products/software/proteinpilot-software) for peptide sequence identification. In some experiments, LC MS was carried out with purified unphosphorylated/phosphorylated histidine-tagged proteins for determining intact mass.

### CD spectroscopy

CD spectra of mGmk (unphosphorylated/phosphorylated) and other point mutant proteins (0.085–0.125 mg/ml, dialysed) were carried out employing a cell with path length of 0.1 cm at 25°C using a Jasco J-810 spectropolarimeter. Each spectrum reported at far UV region (250–198 nm) is an average of four scans and the mean residue ellipticity (θ) was calculated considering 106 Da as the mean of amino acid residue molecular mass.

### Bioinformatics analysis

Primer designing and restriction site identification were done on Gene Runner. Homology searches were performed using BLAST [28,29]. Multiple sequence alignments were carried out to examine conservation of residues using Clustal Omega program [30] available at http://www.ebi.ac.uk/.

### Homology modelling

The open conformation of mGmk was taken from Protein Data Bank (PDBID-1S4Q). The phosphorylated open structure and ATP- and GMP-bound closed structure of this protein were not available in Protein Data Bank. Thus, the open phosphorylated structure was generated by addition of phosphate group to Thr^169^ using Discovery Studio [31]. The closed structure of mGmk has been predicted using homology modelling employing MODELLER [32] based on the closed structure of mouse guanylate kinase (PDBID-1LVG) [33] having ADP in the ATP-binding pocket. Amino acid sequences of these proteins have 43% identity. The position of ATP in modelled closed guanylate kinase was determined by the position of ADP in mouse guanylate kinase. Further, γ phosphate was added to convert ADP into ATP in this closed structure.

### Molecular dynamics simulations

Molecular dynamics (MD) simulations of open and closed structures of mGmk were performed using AMBER14 [34] employing ff14SB force field. The force field parameter for ATP was taken from AMBER parameter database (http://research.bmh.manchester.ac.uk/bryce/amber) and for GMP, these were generated by ANTECHAMBER [35]. To set up the initial structures for MD simulations, leap module of AMBER14 was used. Explicit MD simulations were carried out in TIP3P water molecules [36] in an octahedral box of size 80 × 80 × 80 Å. Appropriate numbers of counter ions Na^+^/Cl^−^ were added to neutralize the net charge. To prevent any steric clashes between solute and solvent, energy minimization was carried out for the solvated system in two steps. In the first step, minimization of water was carried out for 10000 cycles of steepest descent followed by 4000 cycles of conjugate gradient along with positional restraint of 50 kcal/mol/Å^2^ on protein and bound ligands ATP and GMP. The whole system was minimized in the second step for 8000 cycles of steepest descent followed by 4000 cycles of conjugate gradient without any restraints. Heating of minimized system from 10 to 300 K was performed for 300 ps using NVT ensemble and applying 5 kcal/mol/Å^2^ positional restraints on protein backbone atoms and heavy atoms of ATP and GMP. Positional restraints were released gradually in the next two steps, each of 150 ps. Further, equilibration for 100 ps and 2.4 ns were performed employing NVT and NPT ensembles respectively. Finally, production MD simulations were carried out at NVT ensemble for 100 ns. Newton equations of motion were integrated for every 2 fs and non-bonded interactions distance cutoff was set to 10 Å for each simulation. PME calculations were made at a distance cutoff of 10 Å with grid spacing of 1 Å. SHAKE algorithm was used to constrain all the bonds involving hydrogen atoms. MD trajectories were analysed by visualization using VMD [37] and calculations of intraprotein interactions: hydrogen bonds and salt bridges were carried out by employing cpptraj module of AMBER14. For hydrogen bond, D-A distance and D-H-A angle cutoff was kept at 3.5 Å and 135° respectively, while salt bridge was calculated at a distance cutoff of ≤4 Å between positively and negatively charged atoms.

## Results and discussion

**Figure 1:**
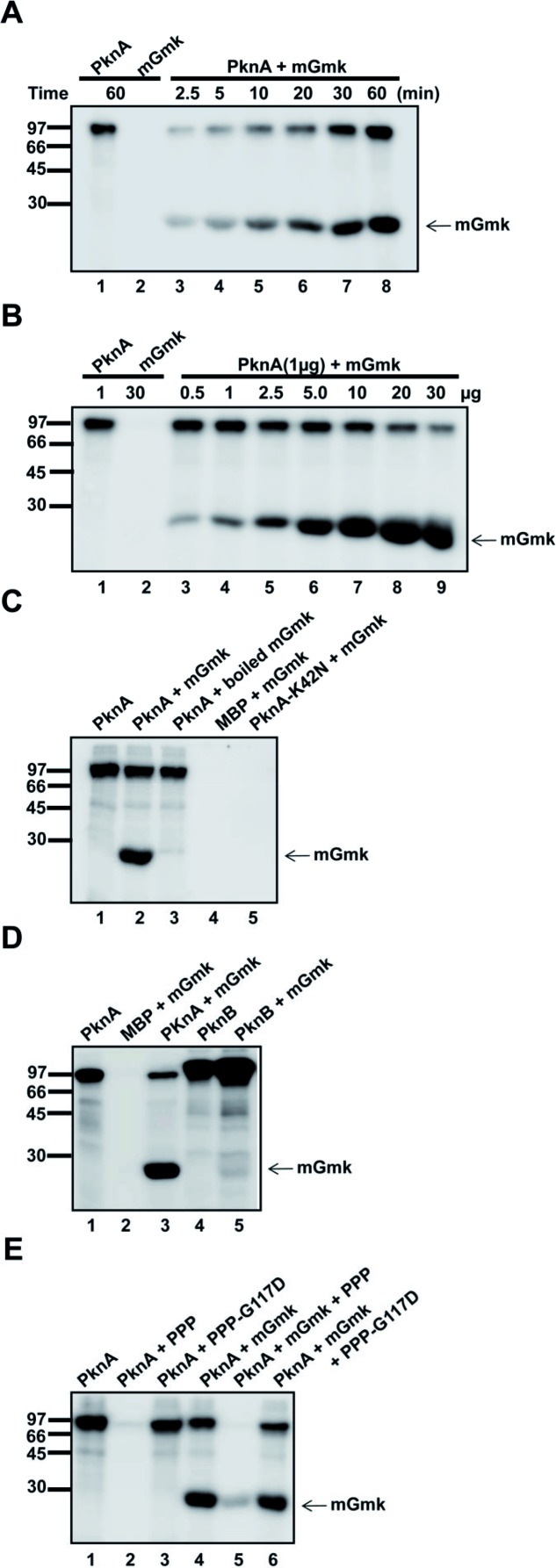
Phosphorylation of mGmk (**A**) Time course of PknA-mediated mGmk phosphorylation. Kinase assay was set up for different time periods (2.5–60 min; lanes 3–8) with mGmk (5 µg/reaction) in the presence of PknA (PknA-core; 1 µg/reaction) and processed as mentioned under ‘Materials and methods’ section ; 60-min incubation with PknA (lane 1) or mGmk (lane 2) served as control. (**B**) Phosphorylation of mGmk as the function of protein concentrations. Kinase assay was carried out for 30 min at 25°C using different amounts of mGmk protein (0.5–30 µg/reaction;) in the presence of PknA (PknA-core; 1 µg/reaction) and processed as mentioned in the text (please see under ‘Materials and methods’ section ). (**C**) Characterization of PknA-mediated phosphorylation of mGmk. Purified mGmk protein was incubated at 90°C for 5 min to heat inactivate the enzyme (boiled) and along with PknA (PknA-core). Also, kinase assay was performed with mGmk protein in the presence of kinase-dead variant of PknA, K42N. (**D**) Phosphorylation of mGmk in the presence of PknB. Kinase assay was performed with mGmk in the presence PknB (1 µg/reaction). (**E**) Effect of PPP on PknA-mediated phosphorylation of mGmk. Kinase assay was performed with mGmk (5 µg/reaction) in the presence of PknA (PknA-core) for 30 min at 25°C and further incubated at 25°C with PPP or G117D (10 µg/reaction) for 60 min. In each figure, numbers in the left indicate protein molecular mass in kilo-Daltons. Also, the position of mGmk is indicated by an arrow and lane numbers are provided at the bottom.

PknA-338 (catalytic and juxtamembrane domains of PknA exhibiting catalytic activity; [22, 38]) and mGmk were purified as MBP- and His-tagged proteins respectively. As shown in Figure 1A, mGmk without PknA in kinase assay did not show any phosphorylation (lane 2). However, its incubation with PknA exhibited phosphorylation and the phosphosignal of mGmk increased as the function of time (Figure 1A, lanes 3–8). The increase in PknA-mediated phosphorylation of mGmk was also observed with increasing amount of protein in a kinase assay (Figure 1B, lanes 3–9). Furthermore, no phosphosignal of mGmk was detected when it was used as a boiled protein (incubated at 96°C for 10 min) in the assay (Figure 1C, compare lanes 2 and 3). This was expected since thermolabile proteins like mGmk would lead to denaturation at high temperature causing loss in transphosphorylation activity. Incubation with a kinase-dead variant, PknA-K42N (Figure 1C, lane 5) also did not show any phosphorylation of mGmk ensuring it is the result of the kinase activity of PknA. Use of PknB, another essential mycobacterial kinase, instead of PknA in the assay yielded weak p-mGmk in kinase assay (Figure 1D, lane 5). To evaluate reversibility of the event, mGmk was incubated with PknA in kinase assay (30 min at 25°C) and this was followed by the addition of PPP, only Ser/Thr phosphatase present in *M. tuberculosis* genome or a dead mutant of the phosphatase, G117D. After incubating for 1 h at 25°C, samples were resolved in SDS/PAGE and processed for autoradiography. While incubation with PPP led to a striking decrease in the level of phosphorylation of mGmk, the G117D had no effect (Figure 1E, lanes 4–6). Thus, all these lines of evidence insinuate the PknA-mediated phosphorylation of mGmk.

**Figure 2:**
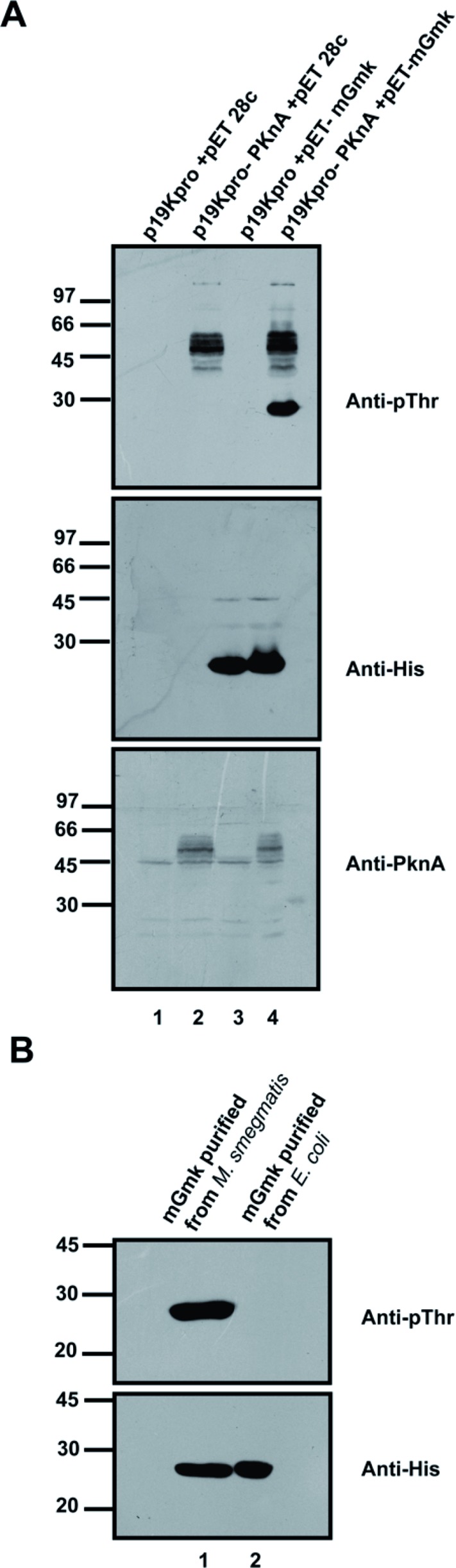
Phosphorylation status of the purified histidine-tagged protein obtained following co-expression of mGmk and PknA (**A**) *E. coli* BL21 (DE3) cells were co-transformed with pET-mGmk and p19kpro-PknA or appropriate vectors. One of the colonies grown in LB agar plates supplemented with both hygromycin and kanamycin was processed for preparation of whole cell lysate. The lysates prepared from colonies (both control and experimental) were resolved in SDS/PAGE (12% gel) and processed for Western blotting using appropriate antibodies (as indicated). (**B**) Phosphoprotein status of mGmk following its expression as a histidine-tagged protein in *M. smegmatis*. His-tagged mGmk protein (1 µg/lane) expressed and purified from *M. smegmatis* (lane 1) and *E. coli* (lane 2) was subjected to Western blotting using anti-p-Thr (upper panel) and anti-His (lower panel) antibodies.

To elucidate whether phosphorylation of mGmk occurs within *in vivo* settings, pET28c/pET-mGmk was co-transformed into *E. coli* strain BL21 (DE3) along with p19kpro/ p19kpro-PknA. A transformant selected over both kanamycin and hygromycin was cultured overnight and cell lysate from this culture was processed for Western blotting using anti-p-Thr, anti-histidine (anti-His) and anti-PknA antibodies. Interestingly, mGmk, when co-expressed with PknA, was recognized by the anti-p-Thr antibody (Figure 2A, lane 4, upper panel) while the same antibody did not recognize mGmk co-expresssed with the vector, p19kpro (Figure 2A, lane 3, upper panel). The presence of PknA and mGmk proteins in cell lysate was ensured by probing with anti-His (Figure 2A, middle panel) and anti-PknA antibodies (Figure 2A, lower panel). Further, to validate whether mGmk is phosphorylated within the mycobacterial system, which has endogenous Ser/Thr kinases, pVV2 vector carrying mGmk was transformed into *M. smegmatis* for expression as a histidine-tagged mGmk protein. The purified mGmk protein from *M. smegmatis* was recognized by anti-p-Thr antibody in Western blotting and His-tagged mGmk purified from *E. coli* was used as a negative control (Figure 2B, upper panel). The same blot was probed with anti-His antibody to confirm loading of the purified proteins. Thus, our results strongly suggest that PknA-mediated phosphorylation of mGmk is not restricted to *in vitro* setup only.

**Figure 3:**
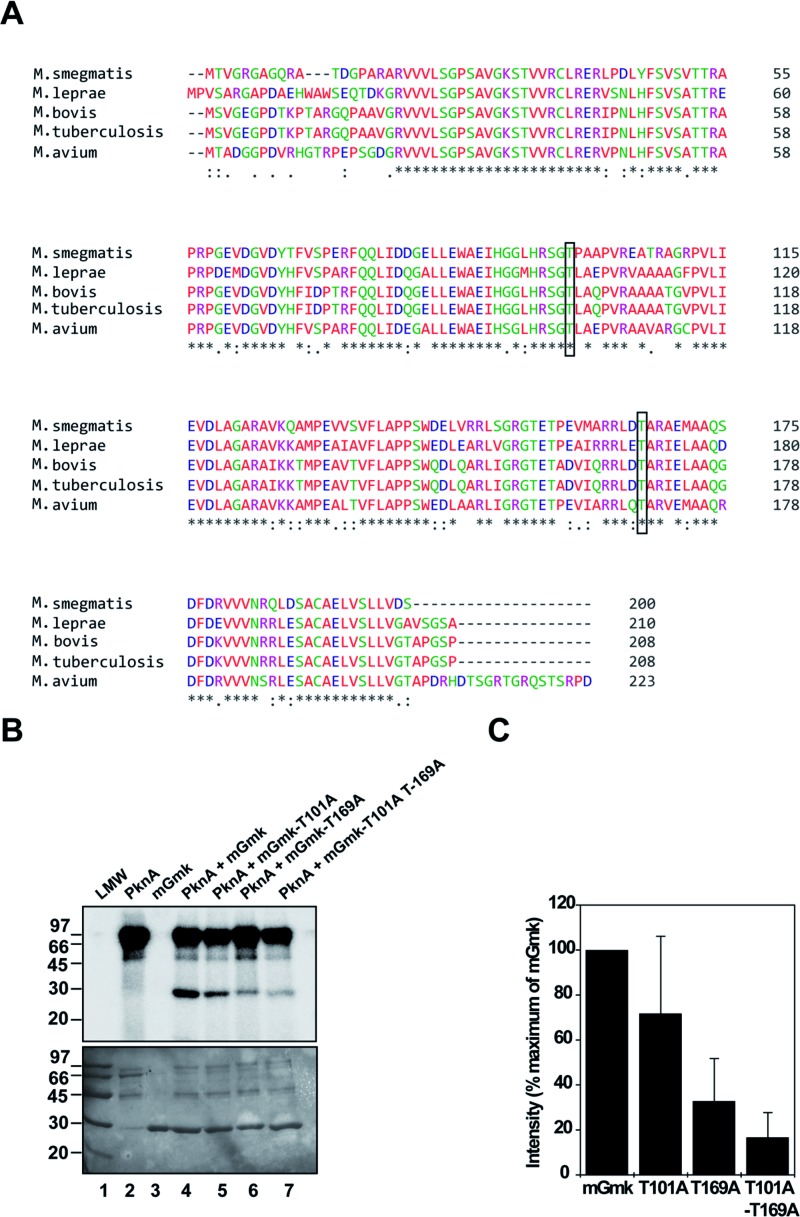
Identification of major phosphosites in mGmk (**A**) Multiple sequence alignment of mGmk and its orthologues from different mycobacterial species. Similar and identical amino acid residues were denoted by dots and asterisks respectively. Numbers indicate position of amino acids in a particular sequence. The residues Thr^101^ and Thr^169^ are shown in boxes. (**B**) Phosphorylation status of mGmK mutant proteins. Two point (T101A and T169A) and one double (T101A-T169A) mutants were generated and assessed for their ability to be phosphorylated in the presence of PknA (PknA-core) in kinase assay (details provided under ‘Materials and methods’ section). Upper panel: autoradiograph; lower panel: same gel used for autoradiograph was stained with Coomassie Brilliant Blue. (**C**) Comparison of phosphorylation intensity of each mutant with the wild-type mGmk protein using ImageJ software [42,43].

To examine the phosphosites within mGmk, the phosphorylated, purified, histidine-tagged protein obtained on co-expression of pET-mGmk/p19kpro-PknA in *E. coli* BL21 (DE3) cells was subjected to trypsin digestion followed by mass spectrometric analysis. The outcome of LC MS/MS revealed that Thr^101^ and Thr^169^ were phosphorylated in our experimental conditions (Table 1 and Supplementary Figure S1). Interestingly, multiple sequence alignment of mGmk orthologues across the mycobacterial species also indicated that Thr^101^ and Thr^169^ are invariant residues (Figure 3A). To assess the contribution of these residues, two point (T101A and T169A) and a double (T101A-T169A) mutants were generated by replacing threonine with alanine. *In vitro* kinase assays with these mutant proteins in the presence of PknA indicated a decrease in phosphorylation of T101A, T169A and T101A-T169A proteins compared with that of the wild-type mGmk (Figure 3B, compare lanes 5, 6 and 7 as opposed to lane 4). However, among the point mutant proteins, the degree of phosphorylation was maximally compromised in T169A (~70%) compared with T101A (~30%). Quantificative analysis of phosphorylation intensity of bands of autoradiographs from three independent experiments utilizing ImageJ software also supported this observation (Figure 3C). In *M. tuberculosis* phosphoproteome mapping, Thr^9^ was identified as the phosphosite in mGmk [11]. To evaluate the contribution of this residue in transphosphorylation of mGmK, we generated a mutant, T9A by altering threonine to alanine. Assessment of its PknA-mediated phosphorylation pattern revealed hardly any difference between T9A (Thr^9^ is absent) and wild-type (Thr^9^ is present) proteins (see Supplementary Figure S2). These observations, therefore, led us to conclude that Thr^169^ is the major phosphosite in mGmk during PknA-mediated phosphorylation. Prisic et al. [11] already predicted phosphorylation motif of mycobacterial eukaryotic-type Ser/Thr kinases as XααααTX(X/V)ϕ(P/R)I where α (−1 position) and ϕ (+3/+5 position) are acidic and large hydrophobic amino acid residues respectively from phosphorylating threonine. Among three phosphosites of mGmk, we noted that Thr^169^ (VIQRRLDT^169^ARIEL), complies with this prediction. While Thr^9^ (SVGEGPDT^9^KPTAR) has acidic residue at −1 position only, the Thr^101^ (GGLHRSGT^101^LAQPVR) does not follow such consensus. Thus, from sequence analysis it seems logical to conclude that Thr^169^ is the preferred phosphorylating amino acid, which is also supported by our experimental evidence (Figures 3B,C).

**Table 1:** Phosphosites in p-mGmk

Peptide	Sequence	Phosphosite(s) in p-mGmk
100–125	GT^*^ LAQPVRAAAATGVPVLIEVDLAGAR	T101
165–182	RLDT^*^ ARIELAAQGDFDK	T169

*Phosphorylated amino acid.

**Figure 4:**
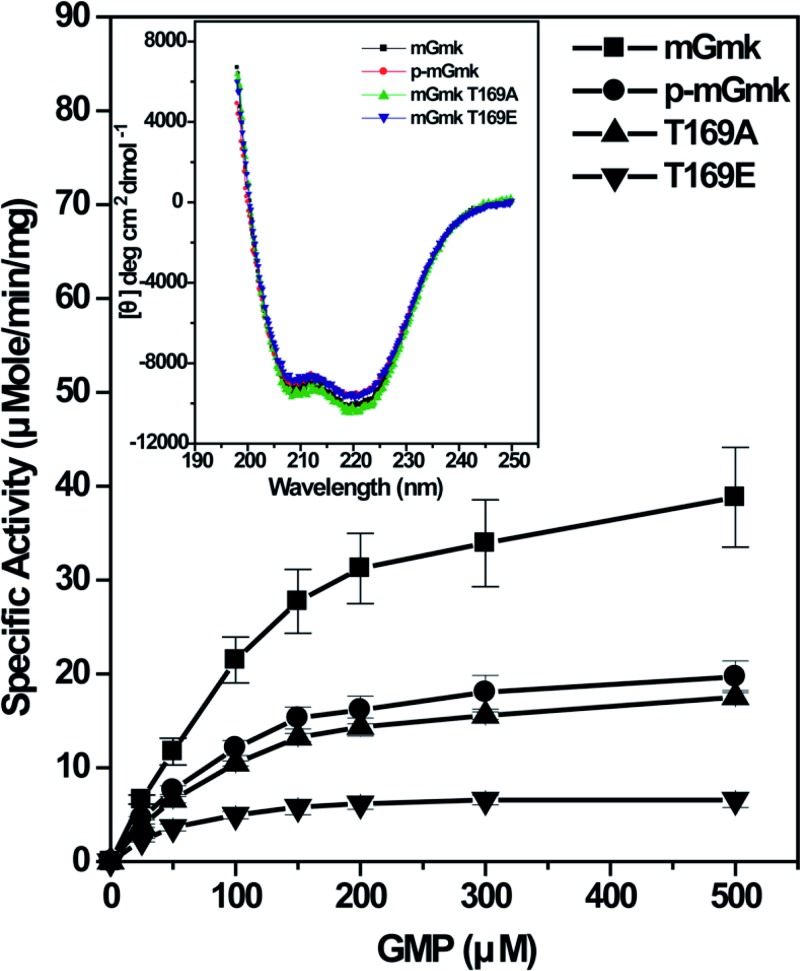
Kinetic analyses of enzymatic activities of mGmk and its variants GDP synthesizing activity of mGmk, its phosphorylated counterpart (p-mGmk) and different mutants (T169A and T169E). Experiments were carried out with different proteins taking indicated concentrations of GMP and 400 µM of ATP. The reproducibility of results was verified in five independent experiments. Inset: CD spectra of indicated proteins used in the present study.

To have an insight into the phosphorylation-mediated modulation of enzymatic activity of mGmk, purified histidine-tagged phosphorylated (p-mGmk) and unphosphorylated proteins were used with increasing concentrations of GMP in microtitre plate based coupled enzymatic assay. Our results indicated that phosphorylation of mGmk resulted decrease in its enzymatic activity (Figure 4). The degree of phosphorylation of p-mGmk was further assessed through intact mass analysis (see Supplementary Figure S3). We observed ~80% in p-mGmk was in phosphorylated form (comprising mono-, di-, tri- and tetrapopulation), while ~20% was in unphosphorylated state. Thus, it was apparent that the decreased enzymatic activity of p-mGmk was the reflection of contribution of ~80% phosphorylated population (Figure 4). Like p-mGmk, the T169A mutant protein (*k*_cat_/*K*_m_ =8 ± 1 × 10^4^ M^−1^ s^−1^) also exhibited decreased enzymatic activity compared with mGmk (*k*_cat_/*K*_m_ =15 ± 2 × 10^4^ M^−1^ s^−1^) indicating a role of Thr^169^ in the enzymatic activity of mGmk (Figure 4 and Supplementary Table S1). Since phosphorylation introduces negative charge into the protein, we assessed the activity of a phosphomimic of Thr^169^ (T169E). As shown in Figure 4, T169E protein also displayed a reduced enzymatic activity (*k*_cat_/*K*_m_ =6 ± 1 × 10^4^ M^−1^ s^−1^) highlighting the contribution of negative charge at Thr^169^ for the reduction in enzymatic activity of mGmk. CD spectra of phosphorylated or mutant proteins also did not show any significant alteration compared with the mGmk, indicating no gross variation in their secondary structures (inset of Figure 4). As expected, we also observed reduction in enzyme turnover rate between T169E (3 ± 0.34 s^−1^) compared with T169A (8 ± 1 s^−1^) signifying the difference in behaviour among the mutant proteins due to the replacement of Thr^169^ with alanine or glutamic acid (Figure 4). It needs to be mentioned here that tri- and tetraphosphorylated forms of mGmk were also detected by intact MS analysis (see Supplementary Figure S3). Such an observation suggests the possibility of phosphorylation of other amino acid(s) in mGmk besides Thr^9^, Thr^101^ and Thr^169^ (Figure 3 and Supplementary Figure S2). Even if there are yet to be identified, other phosphorylating residue(s), the involvement of Thr^169^ in phosphorylation-mediated modulation of mGmk enzyme activity seems to be quite obvious from our results.

**Figure 5:**
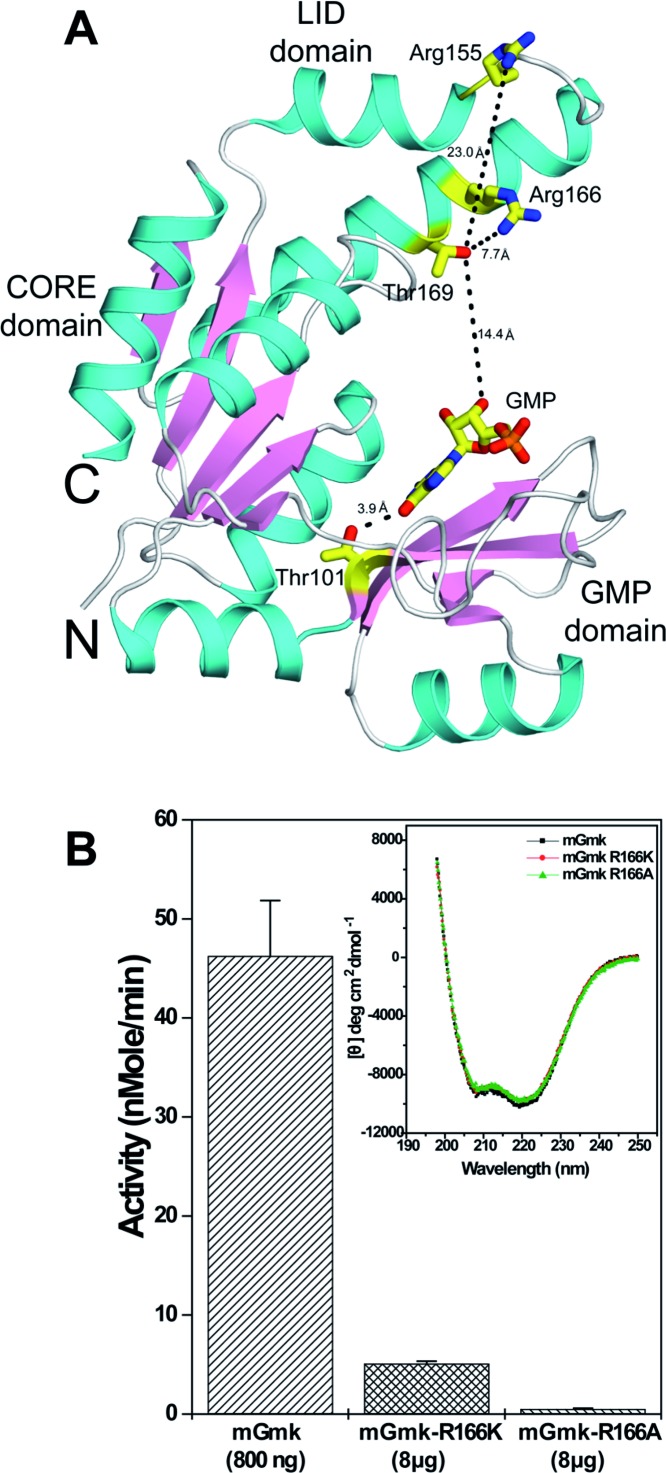
Analysis of Thr^169^ position within mGmk crystal structure (**A**) Cartoon diagram showing the C^α^ tracing of mGmk (generated using the PDBID: 1ZNX). The secondary structure α-helices, β-strands and random structures are shown in cyan, pink and white respectively. The distances are shown in dashed lines. The figure was generated using PyMOL [41]. (**B**) Arg^166^ is crucial for the enzymatic activity of mGmk. Histdine-tagged wild-type and mutant (R166A or R166K) proteins following purification were assessed for their enzymatic activities. Inset: CD spectra of wild-type and mutant proteins.

To understand the contribution of p-Thr^169^ in modulating the enzymatic activity, we investigated the crystal structure of mGmk available in PDB [26]. The mGmk structure complexed with GMP (PDBID: 1ZNX) revealed three major domains namely CORE, LID and GMP binding domain [26]. In the crystal structure, GMP molecule binds in the GMP binding domain and Thr^101^ is located at a distance of 3.9 Å to the bound GMP (Figure 5A). Therefore, it is conceivable that phosphorylation of Thr^101^ may influence GMP binding. On the other hand, Thr^169^ is present in the LID domain and located at a distance of ~14 Å from the bound GMP. This was of little surprise since the phosphorylation of Thr^169^ may not affect the GMP binding directly as it is located too far for any interaction with the GMP. However, for transfer of the phosphate from ATP to GMP, domains of mGmk come close to each other to form a transition state in the presence of ATP and GMP [26]. In fact, earlier reports indicated that two residues, Arg^155^ and Arg^166^ of LID domain are involved in stabilizing the phosphoryl transfer transition state [26]. Strikingly, Thr^169^, the major phosphosite identified in the present study is placed in LID, the region close to P-loop/ATP-binding domain and is present at a distance of 7.7 Å from Arg^166^, while Arg^155^ is placed at a far off distance of ~23 Å. These distance measurements are in the absence of any phosphorylation. Therefore, it is possible that phosphorylation of Thr^169^ might affect the formation of Arg^166^-mediated transition state of mGmk, which is crucial for its functionality (Figure 5A). Thus, to examine the importance of Arg^166^ towards mGmk activity, this residue was replaced with alanine or lysine and the generated mutant was analysed for its activity. Expectedly, both R166K and R166A mutant proteins exhibited a drastic loss in activity though the decrease was more in the latter (Figure 5B). CD analysis of these mutant proteins indicated no significant change compared with wild-type mGmk (inset Figure 5B). To understand the mechanistic details of interaction between p-Thr^169^ and Arg^166^, we performed the MD study of mGmk.

Root mean square deviations (RMSD) of protein backbone atoms were calculated for all the three structures (open, open phosphorylated and modelled closed structure of guanylate kinase) throughout 100-ns trajectory (Supplementary Figure S2). The RMSD of open conformation of mGmk fluctuates around 1.5 and 1.8 Å as observed in two independent MD trajectories. In phosphorylated open conformation of guanylate kinase, RMSD averages around 1.6 and 2.0 Å, while it is 2.0 Å in closed ligand bound kinase during MD trajectories. The values of RMSDs of different conformations of protein show that they remain stable throughout the MD trajectories.

**Figure 6:**
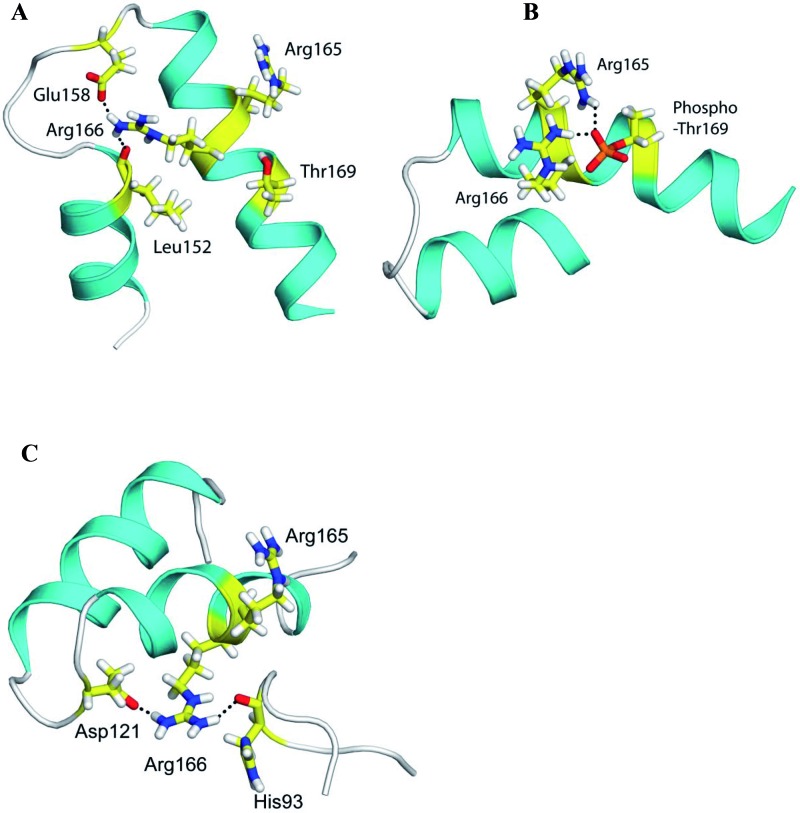
Effect of Thr^169^ phosphorylation on conformation of mGmk Interactions of (**A**) Arg^166^ with Leu^152^ and Glu^158^ in unphosphorylated, open conformation. (**B**) Arg^165^ and Arg^166^ with p-Thr^169^ in phosphorylated, open conformation. Side chain of LID domain residues are shown in stick representation. (**C**) Inter residue interactions in ligand bound closed conformation of mGmk. Interactions among Arg^166^ with His^93^ and Asp^121^. Side chain residues are shown in stick representation.

MD trajectories of phosphorylated and unphosphorylated structures of mGmk have been analysed for hydrogen bond and salt bridge interactions among amino acid residues. In unphosphorylated open conformation of mGmk, Arg^166^, an amino acid residue of LID domain makes hydrogen bonds with backbone carbonyl of Leu^152^, side chain atoms of Thr^169^ and is also involved in formation of salt bridges with Glu^173^, another LID domain residue and Glu^158^ throughout the trajectory with occupancies 83 and 70% and 58 and 69% respectively during MD trajectories (Figure 6A). Phosphorylated open conformation of mGmk triggers Arg^165^ and Arg^166^ (Figure 6B) to make salt bridges with the phosphate group of p-Thr^169^ for 70–80% of total simulation time. Further, analysis of MD trajectory of closed ligand bound mGmk structure revealed that Arg^166^ makes a hydrogen bond with His^93^, a residue of GMP domain (Figure 6C) and a salt bridge with Glu^158^ showing 60–80% occupancy, in one of the MD trajectories. Arg^166^ is also likely to make salt bridge with Asp^121^, an LID domain residue and hydrogen bonds with residues of GMP domain, His^93^ and Gly^94^ with occupancies 18–27% as shown in another MD trajectory of this structure.

The structural analysis of all three conformations of guanylate kinase of *M. tuberculosis* employing MD simulations unravels the effect of phosphorylation of Thr^169^ on the protein conformation. Interactions (hydrogen bond and salt bridge) of Arg^166^ are likely to occur with negatively charged amino acid residues such as Glu^158^ and Glu^173^ or with backbone atoms of Leu^152^ and side chain of Thr^169^ in open conformation of unphosphorylated mGmk as observed during different MD trajectories. However, the residues, Arg^165^ and Arg^166^ make interactions with p-Thr^169^ only during repeated MD simulations of p-mGmk. Experimental data show that there is a decrease in kinase catalytic activity upon phosphorylation of Thr^169^. Thus, it is quite likely that Arg^165^ and Arg^166^ making salt bridges with p-Thr^169^ will not be available to interact with amino acid residues such as His^93^ and Gly^94^ and Asp^121^ and Glu^158^, as observed in ATP and GMP bound closed structure of mGmk. The former couple of residues along with Arg^165^, Arg^166^ and Thr^169^ (residues of LID domain) are involved in maintaining the closed conformation of mGmk as shown in studies by Delalande et al. [39]. Unphosphorylated guanylate kinase upon binding to ligands ATP and GMP allows enzyme to attain closed conformation required for catalysis [39]. However, after phosphorylation of Thr^169^, Arg^165^ and Arg^166^ will form salt bridge with p-Thr^169^ thus, making these unavailable for interactions with Glu^158^, Asp^121^ and residues of loop in GMP domain. This will ultimately affect its catalytic function and thereby decrease in enzyme activity.

Finally, it is imperative to mention at this juncture that inhibition of guanylate kinase activity by (p)ppGpp is associated with bacterial survival in stressed condition [5]. Although such modulation of enzyme activity is evident in most of the actinobacteria, a recent report indicated that (p)ppGpp does not inhibit mGmk activity [40]. In this scenario, it is tempting to speculate that eukaryotic-type Ser/Thr kinase mediated control of mGmk activity is a viable alternative, especially for *M. tuberculosis*, which is very successful in coping up with the stress within the host during infection. Nonetheless, our study convincingly established that Thr^169^ of mGmk being a residue at a distance to its catalytic centre upon phosphorylation affects its enzymatic activity.

## Supporting information

**Figure S1.** LC-MS/MS analysis of phosphorylated mGmk. Fragmentation spectrum for each modified peptide for identification of phosphorylated residues (A and B) following LC-MS/MS are presented (‘b’ and ‘y’ ions are displayed in green).

**Figure S2.** Trans-phosphorylation of mGmk-T9A mutant protein by PknA (PknA-core). The mutant was generated by PCR mediated site-directed mutagenesis approach as mentioned in the text. The mutant histidine-tagged protein was obtained following its cloning in pET28c, transformation of the construct in *E. coli* BL21(DE3) cells and purification through Ni-NTA column as mentioned under ‘Materials and methods’. Trans-phosphorylating ability of this protein by PknA was assessed in kinase assay. Upper panel: Autoradiograph; Lower panel: Coomassie brilliant blue stained gel used for autoradiograph.

**Figure S3.** Quantitation of phosphorylated mGmk by intact mass analysis. Approximately 20% un-phosphorylated mGmk and 80% of mGmk in phosphorylated form (nearly 20% singly, 25% doubly, 25% triply and 10% tetra phosphorylated). A. Intact mass spectra of mGmk and B. Intact mass spectra of p-Gmk

**Figure S4.** Root mean square deviations of backbone atoms of mGmk w.r.t minimized structure during 100 ns MD simulation (duplicate runs in red and green) for A). Open B). Open phosphorylated and C). Ligand bound closed conformation of protein.

**Table S1.** Kinetic analysis of mGmk and its variants

## Abbreviations

MD: molecular dynamics
mGmk: guanylate kinase from *Mycobacterium tuberculosis*
RMSD: root mean square deviation
TBST: TBS supplemented with 0.1% Tween 20
