# Supplementary material for: Eukaryotic-type serine/threonine kinase mediated phosphorylation at Thr169 perturbs mycobacterial guanylate kinase activity
Source: Biosci Rep. 2017 Nov 15;37(6):BSR20171048. doi: 10.1042/BSR20171048 (PMC5686395; doi:10.1042/BSR20171048)
Supplement: Supplementary file 1 [file bsr20171048_Supp1.pdf]

## SUPPLIMENTARY MATERIALS

# Eukaryotic-type Serine/Threonine kinase mediated phosphorylation at Threonine-169 perturbs mycobacterial guanylate kinase activity

**Ghanshyam S Yadav, Sandeep K Ravala, Sangita Kachhap, Meghna Thakur, Abhishek Roy, Balvinder Singh, Subramanian Karthikeyan and Pradip K Chakraborti*#**

CSIR-Institute of Microbial Technology, Sector 39A, Chandigarh 160 036, India

**Short Title**

Phosphorylation affects mGmk activity

*Correspondence to: Pradip K Chakraborti; E-mail: pradip@imtech.res.in;
Telephone: +91 172 2690751; Fax: +91 172 2690585

#Present address: Dept. of Biotechnology, School of Chemical and Life Sciences, Jamia Hamdard (Hamdard University), Hamdard Nagar, New Delhi, 110062, India.
E mail: pkchakraborti@jamiahamdard.ac.in; Alternate E mail:pradipkchakraborti@gmail.com
Telephone: +91-11-26059668 extn. 5583; Cell: +91-9815863975

**A**

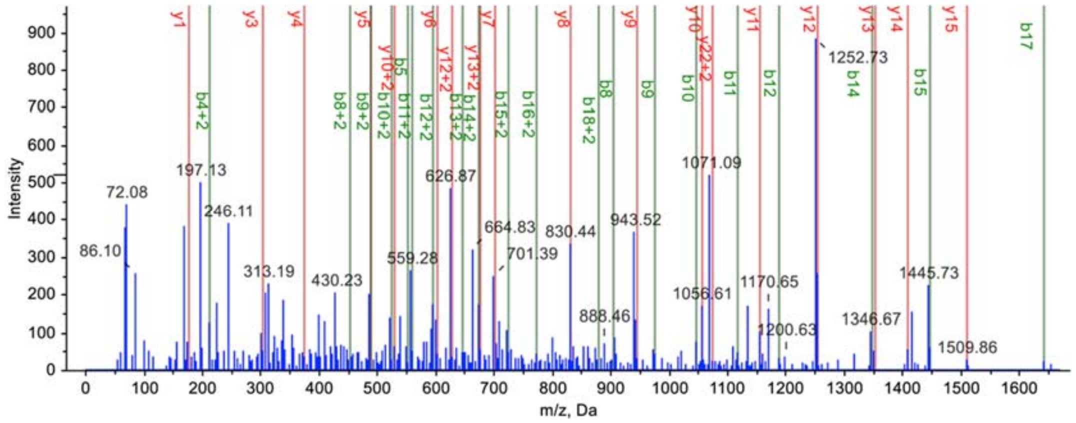


| Residue | b | b+2 | y | y+2 |
|---|---|---|---|---|
| G | 58.0287 | 29.518 | 2697.434 | 1349.22 |
| T[Pho] | 239.0427 | 120.025 | 2640.412 | 1320.71 |
| L | 352.1268 | 176.567 | 2459.398 | 1230.203 |
| A | 423.1639 | 212.0856 | 2346.314 | 1173.661 |
| Q[Dea] | 552.2065 | 276.6069 | 2275.277 | 1138.142 |
| P | 649.2593 | 325.1333 | 2146.234 | 1073.621 |
| V | 748.3277 | 374.6675 | 2049.182 | 1025.094 |
| R | 904.4288 | 452.718 | 1950.113 | 975.5602 |
| A | 975.4659 | 488.2366 | 1794.012 | 897.5096 |
| A | 1046.503 | 523.7552 | 1722.975 | 861.9911 |
| A | 1117.54 | 559.2737 | 1651.938 | 826.4725 |
| A | 1188.577 | 594.7923 | 1580.901 | 790.954 |
| T | 1289.625 | 645.3161 | 1509.864 | 755.4354 |
| G | 1346.646 | 673.8268 | 1408.816 | 704.9116 |
| V | 1445.715 | 723.361 | 1351.794 | 676.4008 |
| P | 1542.768 | 771.8874 | 1252.726 | 626.8666 |
| V | 1641.836 | 821.4216 | 1155.673 | 578.3402 |
| L | 1754.92 | 877.9637 | 1056.605 | 528.806 |
| I | 1868.004 | 934.5057 | 943.5207 | 472.264 |
| E | 1997.047 | 999.027 | 830.4367 | 415.722 |
| V | 2096.115 | 1048.561 | 701.3941 | 351.2007 |
| D | 2211.142 | 1106.075 | 602.3257 | 301.6665 |
| L | 2324.226 | 1162.617 | 487.2987 | 244.153 |
| A | 2395.263 | 1198.135 | 374.2146 | 187.611 |
| G | 2452.285 | 1226.646 | 303.1775 | 152.0924 |
| A | 2523.322 | 1262.165 | 246.1561 | 123.5817 |
| R | 2679.423 | 1340.215 | 175.119 | 88.0631 |

**B**

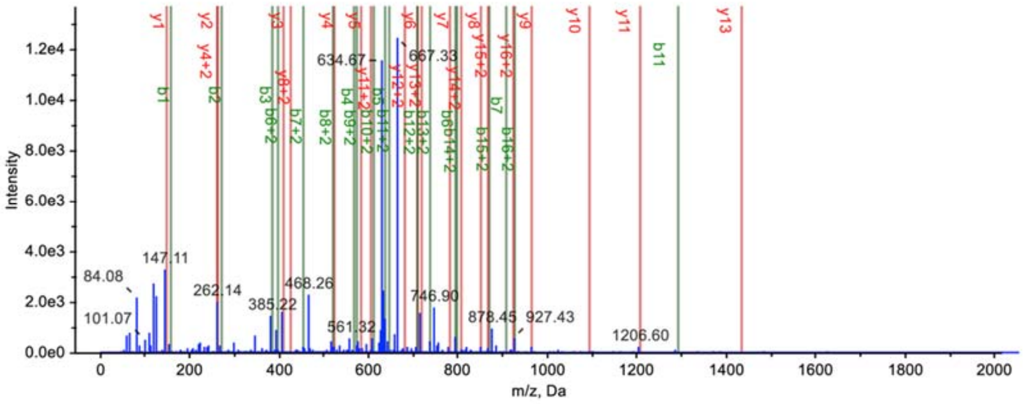


| Residue | b | b+2 | y | y+2 |
|---|---|---|---|---|
| R | 157.1084 | 79.0578 | 1998.964 | 999.9859 |
| L | 270.1925 | 135.5999 | 1842.863 | 921.9353 |
| D | 385.2194 | 193.1133 | 1729.779 | 865.3933 |
| T[Pho] | 566.2334 | 283.6203 | 1614.752 | 807.8798 |
| A | 637.2705 | 319.1389 | 1433.738 | 717.3728 |
| R | 793.3716 | 397.1895 | 1362.701 | 681.8542 |
| I | 906.4557 | 453.7315 | 1206.6 | 603.8037 |
| E | 1035.498 | 518.2528 | 1093.516 | 547.2617 |
| L | 1148.582 | 574.7948 | 964.4734 | 482.7404 |
| A | 1219.62 | 610.3134 | 851.3894 | 426.1983 |
| A | 1290.657 | 645.8319 | 780.3523 | 390.6798 |
| Q | 1418.715 | 709.8612 | 709.3151 | 355.1612 |
| G | 1475.737 | 738.3719 | 581.2566 | 291.1319 |
| D | 1590.764 | 795.8854 | 524.2351 | 262.6212 |
| F | 1737.832 | 869.4196 | 409.2082 | 205.1077 |
| D | 1852.859 | 926.9331 | 262.1397 | 131.5735 |
| K | 1980.954 | 990.9806 | 147.1128 | 74.06 |

**Figure S1**. LC-MS/MS analysis of phosphorylated mGmk. Fragmentation spectrum for each modified peptide for identification of phosphorylated residues (A and B) following LC-MS/MS are presented (‘b’ and ‘y’ ions are displayed in green).

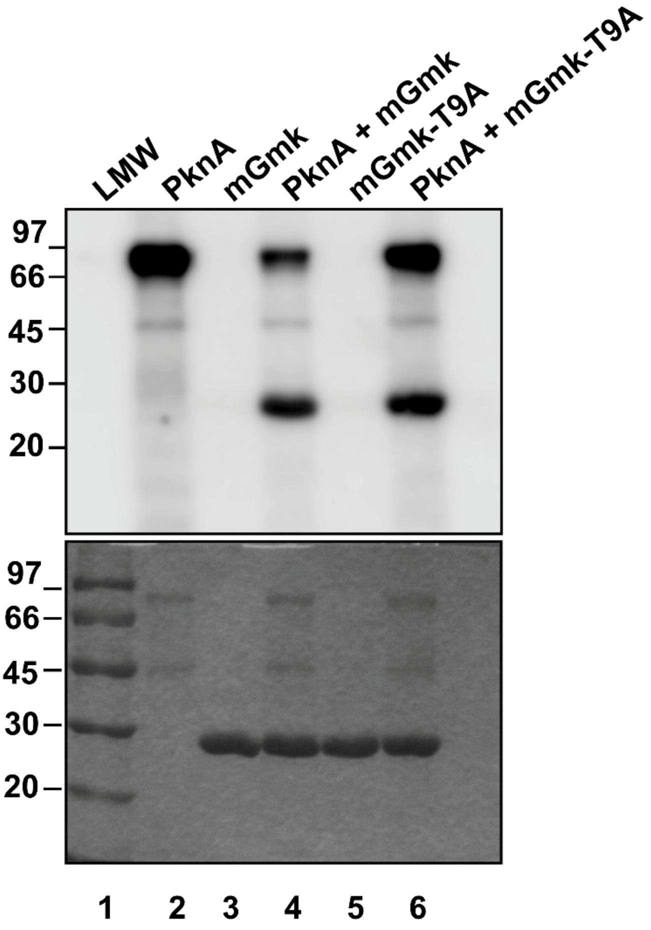


**Figure S2.** Trans-phosphorylation of mGmk-T9A mutant protein by PknA (PknA-core). The mutant was generated by PCR mediated site-directed mutagenesis approach as mentioned in the text. The mutant histidine-tagged protein was obtained following its cloning in pET28c, transformation of the construct in *E. coli* BL21(DE3) cells and purification through Ni-NTA column as mentioned under 'Materials and methods'. Trans-phosphorylating ability of this protein by PknA was assessed in kinase assay. Upper panel: Autoradiograph; Lower panel: Coomassie brilliant blue stained gel used for autoradiograph.

A

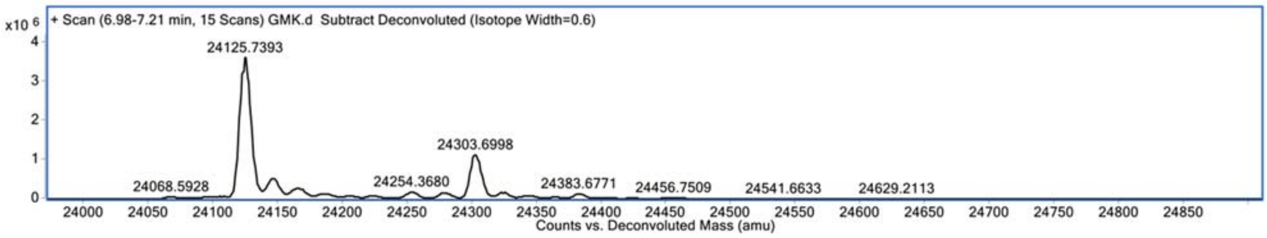


B

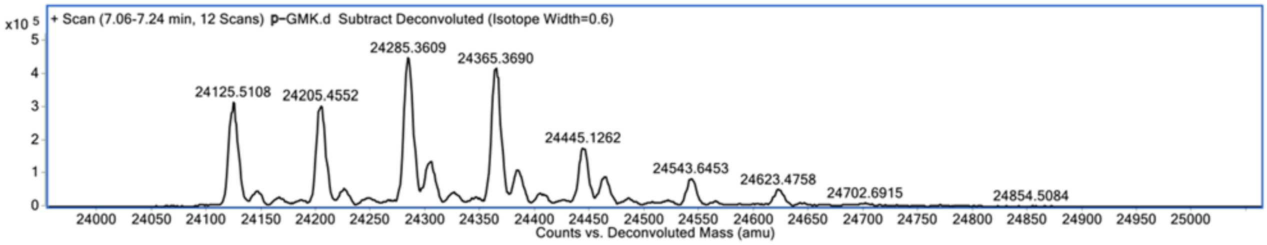


**Figure S3.** Quantitation of phosphorylated mGmk by intact mass analysis. Approximately 20% un-phosphorylated mGmk and 80% of mGmk in phosphorylated form (nearly 20% singly, 25% doubly, 25% triply and 10% tetra phosphorylated). A. Intact mass spectra of mGmk and B. Intact mass spectra of p-Gmk

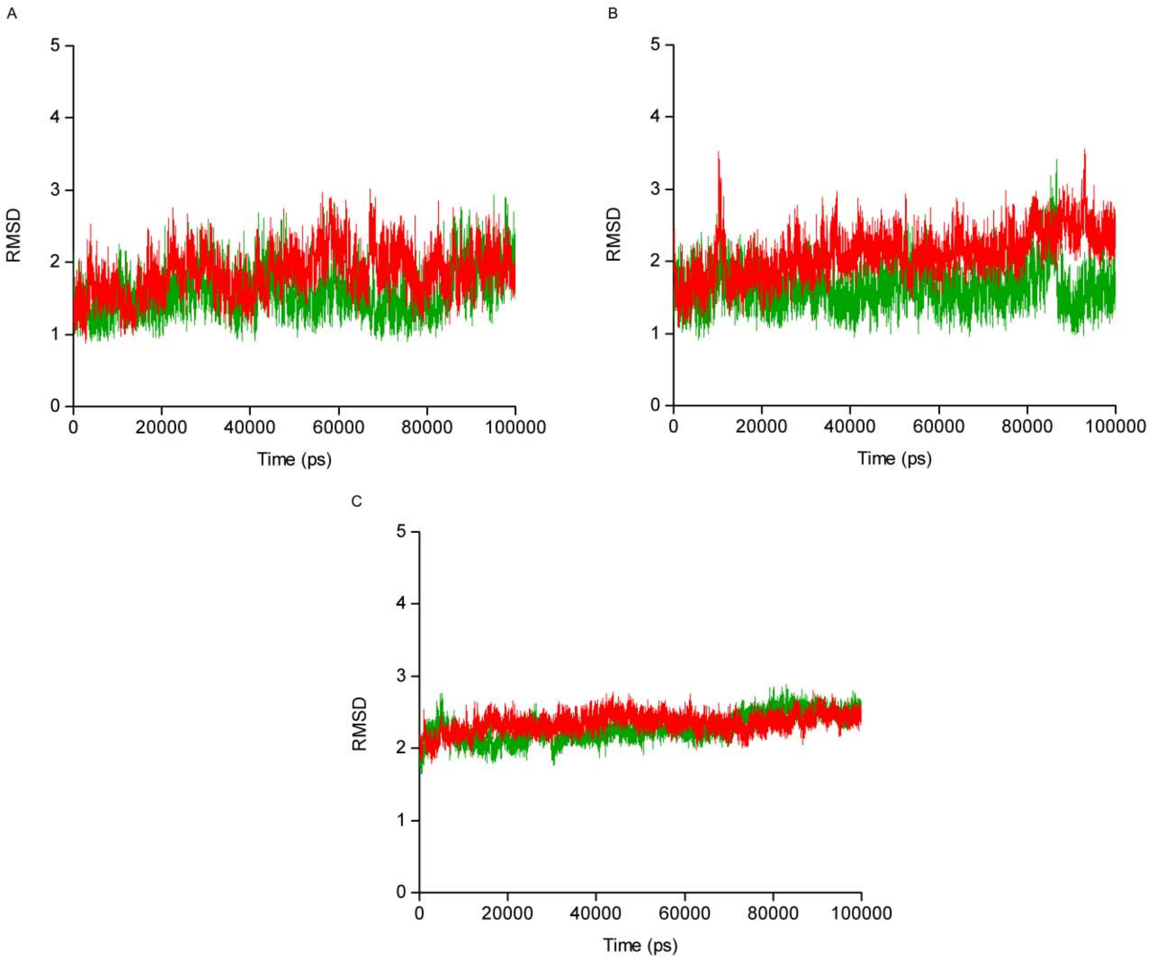


**Figure S4**. Root mean square deviations of backbone atoms of mGmk w.r.t minimized structure during 100 ns MD simulation (duplicate runs in red and green) for A). Open B). Open phosphorylated and C). Ligand bound closed conformation of protein.

**Table S1. Kinetic analysis of mGmk and its variants**

| Protein | $K_m$ (µM) | $k_{cat}$ ($s^{-1}$) | $k_{cat}/K_m$ (x $10^4 M^{-1} s^{-1}$) |
|---|---|---|---|
| mGmK | 138 ± 23 | 20 ± 3 | 15 ± 2 |
| 169A | 99 ± 14 | 8 ± 1 | 8 ± 1 |
| 169E | 53 ± 5 | 3 ± 0.34 | 6 ± 1 |
